# Intraocular Lens-Shell Technique: Adjustment of the Surgical Procedure Leads to Greater Safety When Treating Dense Nuclear Cataracts

**DOI:** 10.1371/journal.pone.0112663

**Published:** 2014-11-17

**Authors:** Lixia Luo, Haotian Lin, Weirong Chen, Bo Qu, Xinyu Zhang, Zhuoling Lin, Jingjing Chen, Yizhi Liu

**Affiliations:** State Key Laboratory of Ophthalmology, Zhongshan Ophthalmic Center, Sun Yat-sen University, Guangzhou, China; Saitama Medical University, Japan

## Abstract

**Objective:**

To compare the efficacy and safety of the intraocular lens (IOL)-shell procedure versus conventional phacoemulsification for the surgical treatment of dense cataracts.

**Methods:**

Eighty eyes with dense nuclear cataracts were enrolled in a prospective, randomized controlled study. Patients were assigned to two groups. In Group I, the IOL was traditionally implanted after all nuclear fragments were completely removed, and in Group II, the IOL was innovatively implanted in the bag before the last residual nuclear fragment was removed. This novel adjusted surgical procedure, named the “IOL-shell technique”, features use of the IOL as a protective barrier rather than simply as a refractive alternative, and it is conceptually different from the traditional step-by-step procedure. Clinical examinations, including uncorrected visual acuity, central corneal thickness (CCT), temporal clear corneal incision thickness and corneal endothelial cell density, were carried out.

**Results:**

The inter-group difference in temporal corneal thickness was found to be of no statistical significance at any of the visits. Compared to eyes in Group I, those in Group II were shown to have significantly less corneal endothelial cell loss on both the 7th and 30th day following surgery. At 7 days after surgery, the mean corneal endothelial cell loss in Group II was 10.29%, compared to 14.37% in Group I (P<0.05). The mean endothelial cell loss measured on postoperative day 30 was 16.88% in Group II compared to 23.32% in Group I (P<0.05). On the 1st day after surgery, the mean CCT of eyes in Group II was significantly smaller compared to Group I (Group I vs. Group II: 19.42% vs. 13.50%, P<0.05).

**Conclusions:**

Compared to conventional phacoemulsification, the IOL-shell technique was shown to be a relatively safer procedure without compromised efficiency for dense cataracts, and it caused less corneal endothelial cell loss and milder postoperative corneal edema (Clinical Trials Identifier: NCT02138123).

**Trial Registration:**

ClinicalTrials.gov NCT02138123

## Introduction

With the development of cataract surgery over recent decades, phacoemulsification with foldable intraocular lens (IOL) implantation has become the first-line treatment for cataracts. [1]–[4] Although phacoemulsification has been shown to be safe, efficient and effective for treating the majority of cataracts, the surgical treatment of dense cataracts with a hard nucleus remains technically challenging. Cataracts with very dense brunescent nuclei are also called “naked-nucleus cataracts” by some clinicians, indicating the nonexistence of a lens cortex between the nucleus and the posterior lens capsule.

In many widely used techniques for conventional phacoemulsification (such as the phaco-chop and divide-and-conquer nuclear fracturing techniques), the nucleus is usually divided first into four to six fragments before being emulsified and removed. The fragmentation and emulsification of the lens nucleus is sequentially followed by removal of the cortical material and in-the-bag IOL implantation. In cases with very dense cataracts, however, the safety and efficacy of phacoemulsification can be greatly compromised due to the lack of protection of the lens capsule by the posterior cortex. [5]–[8] Recently, a common complication has been rupture of the posterior capsule by phaco tips, sharp, hard lens fragments or intense ultrasound waves. Therefore, modified phacoemulsification techniques for treating dense cataracts have been introduced. These new techniques, such as the soft-shell technique combined with a phaco chop, stop-and-chop or drill-and-crack, were shown to improve the safety profile of cataract surgery. [9]–[11] However, due to the lack of protection of the posterior capsule by the posterior cortical layer, posterior capsule rupture still occurs regularly during the surgical treatment of dense cataracts. For this same reason, the emulsification of the very last piece of nuclear fragment is usually the most technically challenging step of the entire procedure. In developing countries, a considerable proportion of cases treated at the cataract service of eye care facilities are dense cataracts. Although extracapsular cataract extraction could be used as an alternative for such cases, phacoemulsification with foldable IOL implantations still provides comparatively better visual outcomes, primarily due to the much smaller incision size.

In this study, we introduce a surgical procedure that we have called the “IOL-shell technique” for the purpose of reducing dense cataract surgery complications, and report a prospective, randomized controlled study to assess the efficiency and safety of the IOL-shell technique, which showed that the new procedure is a safer and equally effective method for hard cataract surgery compared to the conventional phacoemulsification procedure.

## Methods

The protocol for this trial and supporting CONSORT checklist are available as supporting information; see Protocol S1 and Checklist S1.

### Patient Selection

**Figure 1:**
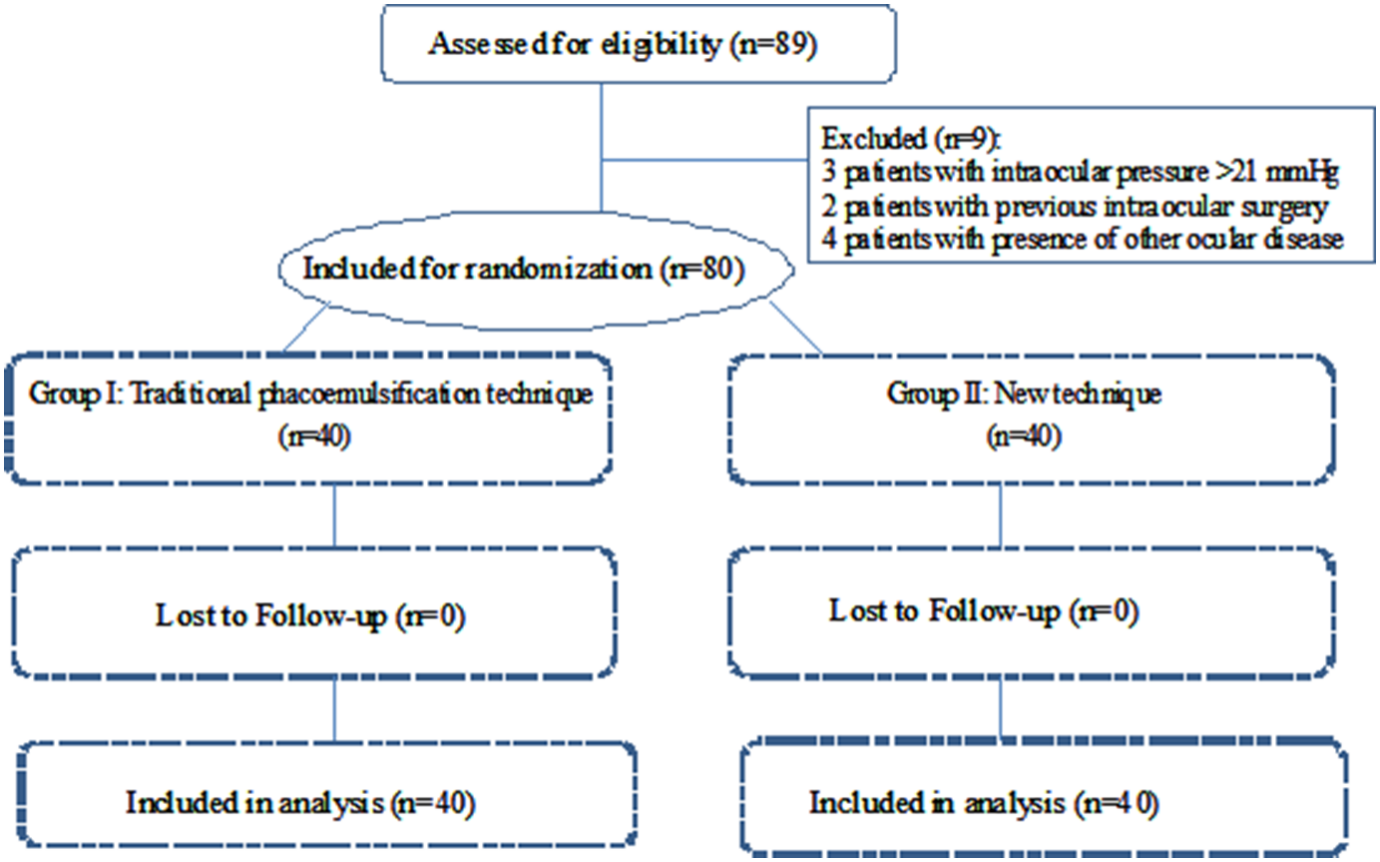
Flowchart of patients included and excluded in the study.

Eighty-nine consecutive patients (89 eyes) identified as having dense cataracts were assessed for eligibility between December 2011 and June 2013 at the Zhongshan Ophthalmic Center (ZOC), Guangzhou, China (Figure 1). Ethical approval was obtained from the Institutional Review Board/Ethics Committee of Sun Yat-sen University. Written informed consent was obtained from each participant. The study was conducted in accordance with the tenets of the World Medical Association’s Declaration of Helsinki. This study was registered with the Clinical Research Internal Management System of ZOC before the enrolment of participants started in order to make this surgical technique confidential. However, when we canceled the plan to apply for a patent of this surgical technique, the study was registered again with ClinicalTrials.gov, NCT02138123, after enrollment of participants started. The authors confirm that all ongoing and related trials for this technique are registered.

### Inclusion and Exclusion Criteria

Inclusion criteria for enrollment included the following conditions: (1) Patients were aged 50 years or more. (2) Dense cataract cases were defined as eyes in which the nuclear color was graded IV or V according to the Lens Opacities Classification System III (LOCS III). Special attention was paid to selecting cases without an apparent posterior cortical layer. (3) There was no central corneal opacification. (4) Pupil diameter was > = 7 mm after full pharmacological dilation during the preoperative assessment. (5) The preoperative central endothelial cell count was > = 1500 cells/mm^2^.

Exclusion criteria for enrollment included any of the following conditions: previous intraocular surgery, abnormal lens zonules, glaucoma, severe myopia (>−6.0 diopters), pseudoexfoliation, uveitis, diabetes mellitus or inability to return to the clinic for follow-up visits.

### Randomization and Grouping

Patients who met the inclusion criteria were randomized into the two groups described above. A random number between 1 and 80, generated by a random number generator, was assigned to each subject. Patients with odd numbers were assigned to Group I (traditional procedure) and those with even numbers were assigned to Group II (novel adjusted surgical procedure). This novel adjusted surgical procedure, the IOL-shell technique, uses the IOL as an intraoperative protective barrier and not just as a refractive alternative, which is conceptually different from the traditional step-by-step procedure. Details of the surgical procedures for both groups are described below.

#### Conventional procedure group (Group I)

In this group, a temporal clear corneal incision was created with a 3.0-mm calibrated knife (Alcon Laboratories). Phacoemulsification was performed with the Infiniti Vision System (Alcon Laboratories) and the Ozil Torsional Handpiece (Micro Tip ABS & Micro Sleeve), which was set to the linear mode in conjunction with ultrasound energy using a phaco chop technique. Nuclear was first divided into four fragmentations using the phaco-chop technique, which was then followed by ultrasound emulsification of the nuclear fragments piece by piece. Due to the lack of a cortical shell within the capsular bag, special care was taken to carry out emulsification of the last nuclear fragment at a relatively more anterior anatomical position between the iris plane and the anterior chamber. A Sensar IOL (AMO Laboratories) was implanted in the capsular bag with the injector system after the lens material was completely removed.

#### IOL-shell technique group (Group II)

In this group, a temporal clear corneal incision was created with a 3.0-mm calibrated knife (Alcon Laboratories). Phacoemulsification was performed with the same device and handpieces and using the same phaco-chop technique as in the conventional procedure group. The difference in the procedure was that before emulsification of the fourth (last) nuclear fragment, cohesive viscoelastic material was injected below the nuclear fragment and gently pushed the fragment to the opposite direction of the temporal corneal incision, then a foldable IOL was implanted into the well-inflated capsular bag posterior to the nuclear fragment. The remaining piece of nuclear fragment was then emulsified and removed within the capsular bag.

### Intraoperative and Postoperative Procedures

**Figure 2:**
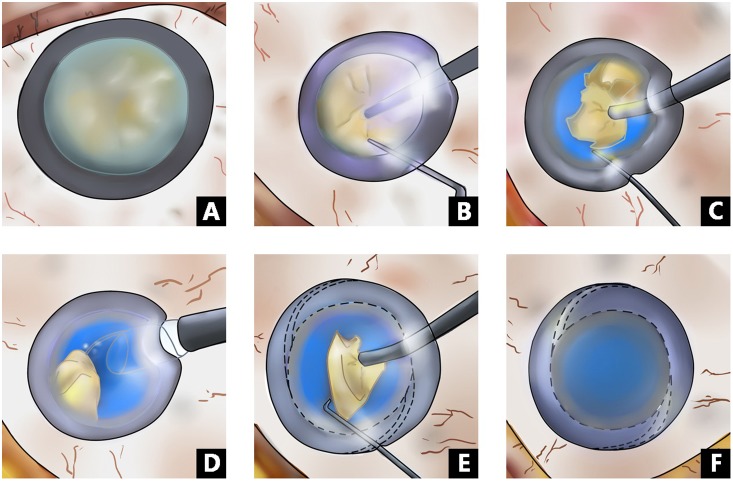
Overview of the IOL-shell technique. **A–F:** A. Hard mature cataract (naked-nucleus), B. The hard nucleus is divided in half with a phaco chop, C. The hard nuclear fragments are emulsified one by one, D. The IOL is inserted between the remaining fragments and the posterior capsule, E. The remaining hard fragments, ready to be emulsified, F. Completion of the surgery.

Topical anesthesia, consisting of a single drop of 0.5% proparacaine (Alcaine, Alcon Laboratories), was administered three times at intervals of 5 minutes prior to surgery. After a temporal clear corneal incision was made, DuoVisc and a soft-shell technique were used to reform and stabilize the anterior chamber and to protect the corneal endothelium. A 5.5–6.0 mm central continuous curvilinear capsulorhexis was created with a bent 26-gauge disposable needle. Hydrodissection was carried out using balanced salt solution (BSS). In Group I, after a standard phaco-chop was performed to remove the lens nucleus completely, an IOL was implanted in the bag, and residual DuoVisc was removed by irrigation and aspiration. In Group II, the nucleus was divided into fragments using a standard phaco-chop. After most of the fragments were removed, the IOL was implanted in the bag before the residual fragment was removed (Figure 2).

No sutures were used to close the clear corneal incision. Postoperative topical therapy included 0.3% tobramycin and 0.1% dexamethasone eye drops (Tobradex, Alcon Laboratories) four times per day and 0.3% tobramycin and 0.1% dexamethasone eye ointment (Tobradex, Alcon Laboratories) every night for one month. All patients returned for follow up visits 1, 7 and 30 days after surgery.

### Outcome Measurements

Principal safety outcomes included four measurements: central corneal endothelial cell loss, central cornea thickness, temporal corneal incision thickness and posterior capsular rupture (PCR). Intraoperative outcomes included five measurements: ultrasound time (UST), US total equivalent power in position 3 (US power), cumulative dissipated energy (CDE), total BSS volume (V_BSS_), and other surgical complications apart from PCR that were recorded at the end of each surgery.

The uncorrected visual acuity (UCVA) was measured and recorded using an Early Treatment Diabetic Retinopathy Study (ETDRS) chart both before surgery and on the 1^st^, 7^th^ and 30^th^ days after surgery. The temporal corneal thickness and central corneal thickness were measured preoperatively and at each postoperative visit using the manual measurement scale in scanned images from anterior segment optical coherence tomography (AS-OCT, Zeiss Meditec, Dulbin, CA, USA). [12] Central corneal endothelial cell density was recorded preoperatively and on the 7^th^ and 30^th^ postoperative days using non-contact specular microscopy (SP2000P, Topcon Tokyo, Japan). Central corneal endothelial cell loss was calculated by subtracting the postoperative corneal endothelial cell density from the preoperative baseline level.

All surgeries were performed by the same experienced surgeon (Y.L.). Intraoperative and postoperative outcomes and complications were observed by two senior ophthalmologists (L.L. and H.L.). The same masked technician (Z.L.) performed AS-OCT for all patients. Both the technician and ophthalmologists were masked to the patients' group assignment during post-operative examinations.

### Statistical Analysis

The SPSS software package (version 17.0, SPSS Inc, Chicago, IL, USA) was used for statistical analysis. Changes of central corneal thickness and changes of temporal corneal incision thickness were compared using repeated measures ANOVA. Mean central cornea endothelial cell loss, UST, US power, CDE, and total BSS volume of the two groups were compared using a t test. Pearson’s chi-squared test was used for comparing gender, nucleus grade, Fisher’s exact test was used for preoperative UCVA and Mann-Whitney test was used for postoperative UCVA. Changes in outcomes were compared between treatments using a mixed effects model with covariate adjustment for baseline outcome. Mean changes and 95%CI derived from the mixed models are presented. All statistical tests were two-tailed with α = 0.05. A p value of <0.05 was considered statistically significant.

## Results

Eighty patients (80 eyes) that met the inclusion criteria were enrolled and randomly allocated into two groups, with 40 patients in each group (Figure 1). All patients completed every follow-up visit. The distribution of age, gender, surgical eye and nuclear cataract grade were comparable between the two groups at baseline (Table 1). Intraoperative complications (including posterior capsular tear, vitreous loss, retained lens fragments and wound burns) were not observed in any of the cases.

**Table 1:** Clinical and demographic data from the subjects in the two groups.

Demographics	Group I(conventional procedure)	Group II(IOL-shell-technique)	*P* value
Patients/Eyes (n)	40/40	40/40	–
Mean age (y) ± SD*	73.55±8.58	72.20±8.08	0.471
Males/females (n)	19/21	22/18	0.502
Right/left eye (n)	20/20	23/17	0.501
**Nuclear density**			
Grade 4 (n/%)	29/72.5	28/70	0.805
Grade 5 (n/%)	11/27.5	12/30	
Mean nuclear density*	4.23±0.42	4.30±0.46	0.452

*t-test, otherwise chi-squared test.

### Outcomes of surgical efficiency

Intraoperative parameters were recorded to assess surgical efficiency, including UST, US power, CDE (one of two principal efficacy outcomes) and total BSS volume. There were no statistically significant differences in measurements of these variables between the two groups (Table 2).

**Table 2:** Comparison of intraoperative parameters measured in the two groups.

Parameters▴	Group I(conventional procedure)	Group II(IOL-shell-technique)	*P* value
UST (sec)	116.57±10.35	115.69±8.19	0.947
US Power (%)	21.02±3.67	21.46±5.11	0.663
CDE (%)	25.68±14.28	25.32±12.03	0.904
V_BSS_ (ml)	76.03±23.59	79.03±24.19	0.576
Complications(n)	0	0	---

UST = ultrasound time; CDE = cumulative dissipated energy; V_BSS_ = total volume of balanced salt solution.

▴ Expressed as mean ± SD.

### Uncorrected visual acuity

After surgery, the mean UCVA was significantly improved compared to baseline levels in both groups. On postoperative day 1, 34 of 40 eyes in Group I (85%) and 32 of 40 eyes in Group II (80%) had UCVA>20/63. On day 7, all eyes had UCVA>20/63. No significant difference was found in UCVA between the two groups at any follow-up time (Table 3).

**Table 3:** Uncorrected visual acuity in the two groups after surgery.

UCVA	Group I(conventional procedure)	Group II(IOL-shell-technique)	*Z*	*P* value
Pre-operation†				0.970
LP (n/%)	1/2.5	1/2.5		
HM (n/%)	27/67.5	28/70		
FC (n/%)	12/30	11/27.5		
Post-operation‡				
day 1	0.3[0.2–0.5]	0.3[0.1,0.5]	0.376	0.707
day 7	0.2[0.05,0.2]	0.2[0,0.2]	0.898	0.369
day 30	0.1[0,0.2]	0.1[0,0.2]	0.808	0.419

All values are mean ± SD.

LP = light perception.

HM = hand move.

FC = finger count.

UCVA = uncorrected visual acuity.

† Fisher’s exact test.

‡ Mann-Whitney test.

### Changes in clear corneal incision thickness and central corneal thickness

**Figure 3:**
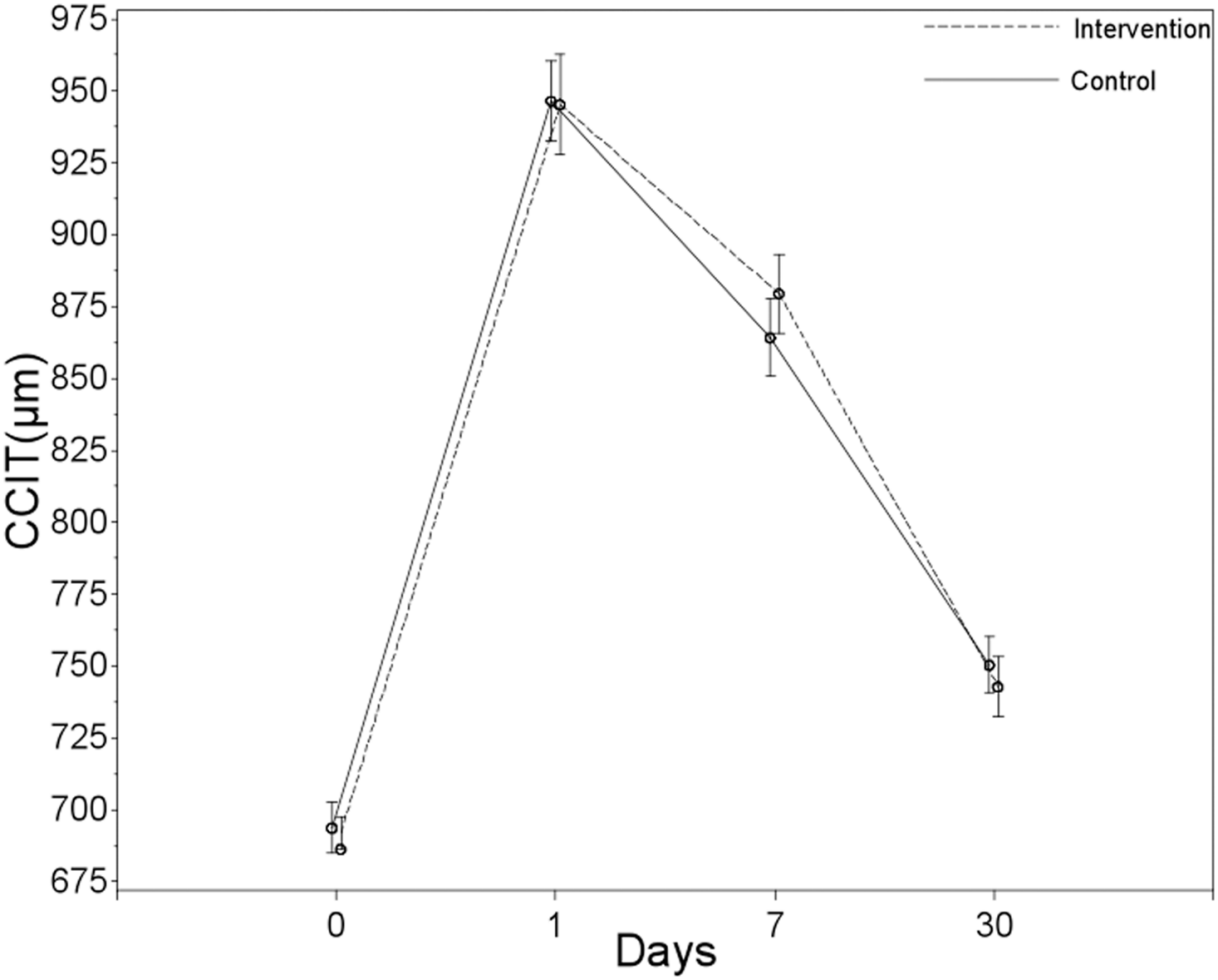
Changes in clear corneal incision thickness (CCIT) between two groups at each visit.

Changes in temporal clear corneal incision thickness (CCIT) and central corneal thickness (CCT) were measured using AS-OCT on the 1^st^, 7^th^ and 30^th^ days after surgery. No statistically significant differences were found in changes in clear corneal incision thickness between the groups for any of the post-operative visits (Table 4, Figure 3).

**Table 4:** Changes in maximal clear corneal incision thickness (µm)and central corneal thickness (µm) for Intervention and Control groups at 1,7 and 30 Days.

		Mean (95% Confidence Interval)			
Outcome	Day	Intervention Group	Control Group	Treatment effect (Intervention-Control)†	Between-Group *P* Value
CCIT	1	267.31[241.76 to 292.87]	254.74[229.18 to 280.29]	12.57[−23.63 to 48.78]	0.4937
	7	201.56[176.01 to 227.12]	172.39[146.83 to 197.94]	29.17[−7.03 to 65.38]	0.1134
	30	64.81[39.26 to 90.37]	28.46[32.91 to 84.02]	6.35[−29.85 to 42.55]	0.7295
CCT	1	73.02[60.51 to 85.53]	101.48[88.97 to 113.99]	−28.47[−46.18 to −10.76]	0.0018
	7	2.27[−10.24 to 14.78]	7.48[−5.03 to 19.99]	−5.22[−22.93 to 12.49]	0.5616
	30	1.77[−10.74 to 14.28]	2.23[−10.28 to 14.74]	−0.47[−18.18 to 17.24]	0.9586

† Treatment effect defined as the change in the intervention group-the change in the control group.

CCIT = clear corneal incision thickness.

CCT =  central corneal thickness.

Changes in maximal CCIT = postoperative maximal CCIT - preoperative CCIT.

Changes in CCT = postoperative CCT - preoperative CCT.

**Figure 4:**
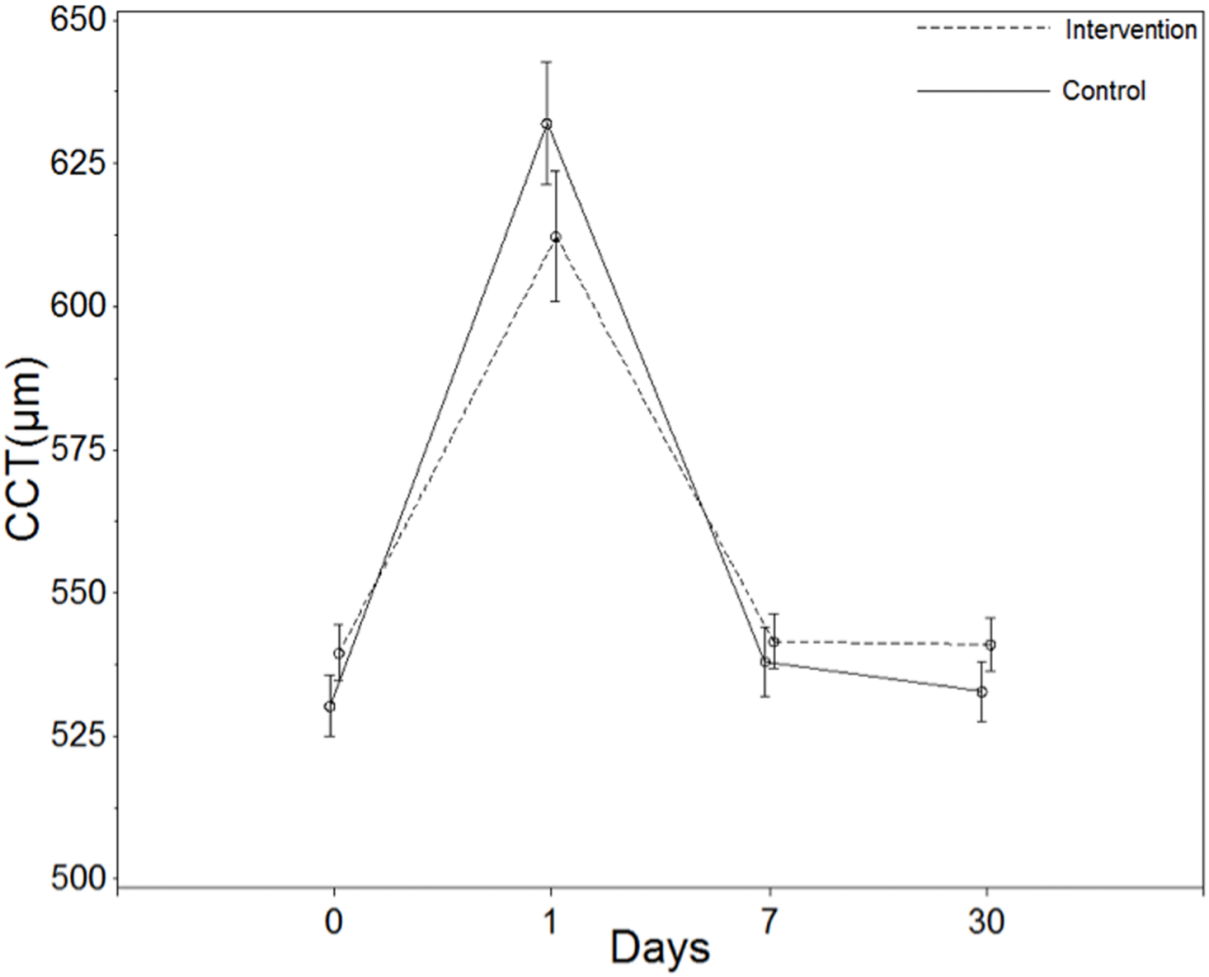
Changes in central corneal thickness (CCT) between two groups at each visit.

On postoperative day 1, patients in Group II had a significantly smaller increase in central corneal thickness than Group I patients (*P*<0.05). At later follow-up visits, the inter-group difference in the change in CCT was no longer statistically significant (Table 4, Figure 4).

### Central corneal endothelial cell loss

**Figure 5:**
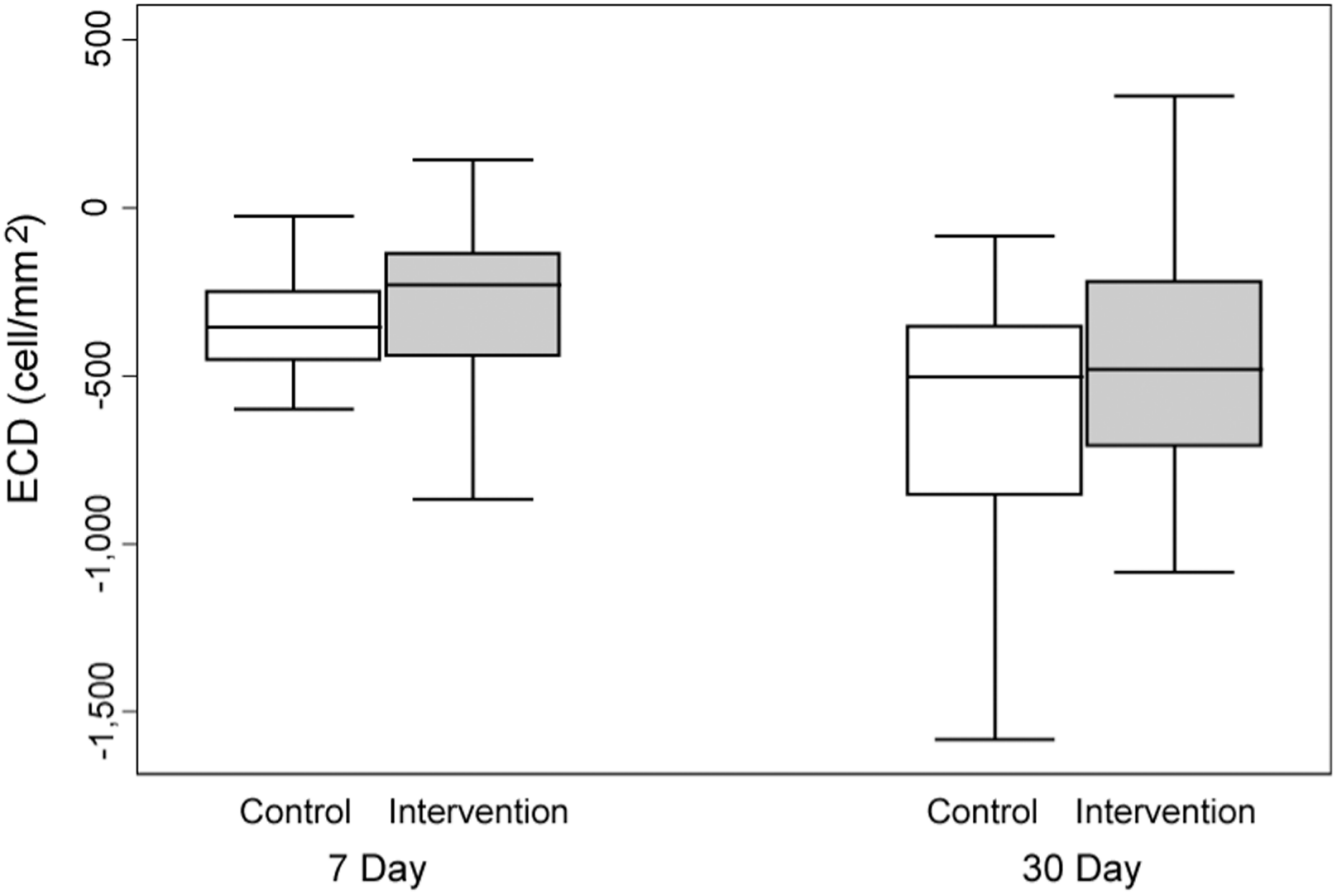
Central corneal endothelial cell loss.

Central corneal endothelial cell loss was recorded on the 7^th^ and 30^th^ days following surgery. On postoperative day 7, the mean central corneal endothelial cell loss in Group II was 10.29% compared to 14.37% in Group I (t = 2.005, p = 0.048). On postoperative day 30, the mean central corneal endothelial cell losses of Group II and Group I were 16.88% and 23.32% (t = 2.003, p = 0.046), respectively. (Figure 5).

## Discussion

In the current study, an innovative method, the IOL-shell technique, was employed for the purpose of protecting the posterior lens capsule during the phacoemulsification of dense brunescent cataracts without posterior cortical layers. We prospectively compared the safety and efficacy of the IOL-shell technique with the conventional phacoemulsification procedure and found significantly less corneal endothelial cell loss and milder central corneal edema.

Dense cataracts, which are relatively more common in developing countries, are generally more technically challenging even for skilled cataract surgeons. During conventional cataract surgeries for cataracts of low or moderate density, the posterior lens capsule is usually well protected by an integral layer of cortex during the emulsification of nuclear fragments to ensure reasonably good safety. Brunescent cataracts with a very bulky and hard nucleus, however, typically pose the most difficulty during surgical treatment due to the extremely thin or nearly non-existent subcapsular cortical layers. In these cases, the posterior capsule can be directly exposed to risk from various factors, such as the phaco tip, sharp, hard nuclear fragments and ultrasound waves. The risk for PCR is especially high when a surge occurs. When the fragmented nucleus is removed piece by piece, the capsular bag gradually loses support and tends to collapse more easily. Any instability of the fluidity during surgery can cause remarkable movement of the posterior lens capsule. PCR can easily occur when the anteriorly displaced posterior capsular accidentally touches the sharp, hard, moving and rotating nuclear fragments, which may be very difficult to control especially when dealing the very last piece of nuclear fragment. To avoid PCR in dense cataracts without a posterior cortical shell, the surgeon often manipulates and emulsifies the last piece of nuclear fragment at a relatively more anterior plane in the eye, which may obviously pose more risk for damaging the corneal endothelium and may also lead to more significant corneal edema after surgery. [13] Despite the introduction of various phaco techniques during recent years, PCR is still a frequent complication of cataract surgeries for dense cataracts. The reported incidence is greater than 5% [14], [15].

The visco-shell technique, which was based on a case series study with a very small sample size, was proposed to protect the posterior capsule in surgeries for cataracts with hard nuclei. [16] The conclusion of this small case series study was not verified by later studies with more stringent designs and larger sample sizes. In the current study, rather than using a layer of viscoelastic material as the protective shell, we used the IOL as a barrier to prevent the posterior lens capsule from being ruptured by the sharp edges of the nuclear fragments or phaco tips. With the protection provided by the IOL that is inserted into the capsular bag before the removal of the last nuclear fragment, surgeons can comfortably manipulate and emulsify the last piece of nuclear fragment within the capsular bag. This protection may also help explain the lower corneal endothelial cell loss and milder corneal edema after surgeries using the IOL-shell technique. In this IOL-shell technique, the IOL can also serve as an ideal mechanical barrier to keep the posterior capsular lens in a stable and safe position and prevent the adverse impact of ultrasound waves or unstable fluidity on the posterior lens capsule.

Based on a number of measurements from the two randomized groups, i.e., the conventional procedure group and our new procedure group, we did not find significant differences in mean UST, US power, CDE and BSS volume between the two groups, indicating that these two techniques are equally efficient. The UCVA results showed no statistically significant differences between the two groups, indicating that both techniques have equally fast restoration of visual function.

Corneal edema was evaluated using clear corneal incision thickness and central corneal thickness measured by high-resolution AS-OCT images. Although the two groups did not show significant differences in clear corneal incisions, Group II patients had significantly less central corneal thickness than Group I patients on day 1. This finding may suggest less corneal endothelial damage with our technique. This observation was further supported by corneal endothelial cell counts on both day 7 and day 30 after surgery.

To summarize, in this prospective, randomized controlled study, phacoemulsification using the IOL-shell technique was shown to be equally effective but relatively safer for dense nuclear cataracts with very little cortex compared to conventional phacoemulsification. This technique may serve as a valuable complement to the soft-shell technique to maximally enhance the safety profile of surgical treatment for dense cataracts.

## Supporting Information

Protocol S1 **The study protocol for this trial.** 1. Background and Purpose, 2. Patient Recruitment and Enrollment, 3. Surgical Protocols, 4. Follow-up and evaluation of surgical outcomes, 5. Statistical Analyses, 6. References.
(DOCX)

Checklist S1 **CONSORT checklist.**
(DOC)
