# Supplementary material for: Intraocular Lens-Shell Technique: Adjustment of the Surgical Procedure Leads to Greater Safety When Treating Dense Nuclear Cataracts
Source: PLoS One. 2014 Nov 17;9(11):e112663. doi: 10.1371/journal.pone.0112663 (PMC4234368; doi:10.1371/journal.pone.0112663)
Supplement: Protocol S1 — The study protocol for this trial. 1. Background and Purpose, 2. Patient Recruitment and Enrollment, 3. Surgical Protocols, 4. Follow-up and evaluation of surgical outcomes, 5. Statistical Analyses, 6. References. (DOCX) [file pone.0112663.s001.docx]

**Intraocular Lens-Shell Technique: Adjustment of the Surgical Procedure Leads to Greater Safety When Treating Dense Nuclear Cataracts**

**Study Protocol**

**Table of Contents**

1. Background and Purpose………………………………………………………………...2

2. Patient Recruitment and Enrollment …………………………………………………….4

3. Surgical Protocols ……………………....……………………………………………….5

4. Follow-up and evaluation of surgical outcomes………………………………………....6

5. Statistical Analyses……………………………………………………………………….7

6. References………………………………………………………………………………. .8

**Chapter 1**

**Background and Purpose**

**1.1 Objectives**

This study is a randomized clinical study with the following objectives:

- To determine the efficacy of phacoemulsification combined with intraocular lens (IOL) implantation by (1) the traditional procedure or (2) our new technique—IOL shell procedure in hard nucleus cataract surgery.
- To determine whether patients with hard nucleus cataract have less surgical complications following cataract surgery by (1) the traditional procedure or (2) our new technique-- IOL shell procedure.

**1.2 Rationale of the Study**

The study is conducted for the following reasons:

- Patients with hard cataract are very common in developing country. However, the application of phacoemulsification in these cases has been hindered by two surgical technical problems:

(1)There is few effective prevention methods for posterior capsular, traditional phacoemulsification techniques in hard nucleus cataract have serious surgical complications such as posterior capsular rupture and fragment dropping in vitreous cavity.

(2) It has been reported that Chinese have significant less anterior chamber depth than Caucasian. In order to avoid the posterior capsule rupture, traditionally, when emulsifying the last piece of fragment, the surgeon will intentionally elevate the nucleus to the anterior chamber, closer to the cornea, which increase damage on the corneal endothelial cells, leading to serious corneal edema.

- In this study, the IOL-shell technique allowed the surgeon to emulsify the last piece of fragment more posterior and comfortably in the bag with no need to worry the rupture of posterior capsule by the tip. Meanwhile, the increased distance between the phaco tip and endothelium provides more space that helps minimize the corneal endothelial damage.
- The advantage of our new technique may become more meaningful particularly in the eyes with endothelial dysfunction or shallow anterior chamber.
- The IOL act as a scaffold in our study provides a possible solution to above-mentioned problems.

**1.3 Summary of Study Protocol**

**Major eligibility criteria:**

- Cataract grade is severe than Grade 4.
- No other eye diseases except cataract.
- Pupil dilation >= 7 mm at the time of preoperative examination.
- Transparent central cornea, and a preoperative central endothelial cell count of >= 1500 cells per square millimeter.

**Treatment groups:** Eligible patients underwent one of two surgical techniques for cataract extraction: a new technique (the IOL was implanted in the capsular bag before the last nuclear fragment was removed) or the traditional technique (the IOL was implanted in the capsular bag after all nuclear fragments were removed).

**Examination Schedule:** The un-corrected visual acuity (UCVA) was recorded, using an ETDRS chart preoperatively at postoperative day 1, 7 and 30. The maximal incision thickness and central corneal thickness were recorded by anterior segment optical coherence tomography preoperatively and at each postoperative visit. Central corneal endothelial cell density was recorded preoperatively and at postoperative 7 and 30 day by non-contact specular microscopy. Central corneal endothelial cell loss was calculated on the basis of preoperative and postoperative endothelial cell density.

**Primary Outcome**: Endothelial cell loss, changes of central and temporal corneal thickness

**Secondary Outcomes:** Visual function, the mean ultrasound time (UST), US total equivalent power in position 3 (US power), the mean cumulative dissipated energy (CDE) and total BSS volume (V_BSS_), incidence of posterior capsular rupture and other ocular complications.

**Chapter 2**

**Patient Recruitment and Enrollment**

**2.1 Participant Recruitment**

Age-related cataract patients meeting with the eligibility criteria were enrolled for the study.

**2.2 Exclusion Criteria**

Patients will be excluded from the study if they meet any of the following criteria:

- Intraocular pressure >21 mmHg
- Previous intraocular surgery
- Presence of other ocular disease (e.g. abnormal lens zonules, keratitis, glaucoma)
- History of ocular trauma

**2.3 Examination Procedures**

Ophthalmic examination will assess:

1. Visual acuity measured by ETDRS chart
2. Slit-lamp examination/photography
3. Anterior segment optical coherence tomography
4. Non-contact specular microscopy
5. Pupil dilation and cataract degree evaluation
6. Non-contact tonometer

Surgical equipment parameters will assess:

1. Ultrasound time (UST)
2. US total equivalent power in position 3 (US power)
3. Cumulative dissipated energy (CDE)
4. Total BSS volume (V_BSS_)

**Chapter 3**

**Surgical Protocols**

**3.1 Treatment Groups**

Patients will be assigned to one of two treatments:

1. Traditional phacoemulsification technique: IOL was implanted in the capsular bag with the injector system after nucleus was completely removed.
2. New technique: IOL was implanted in the capsular bag before the last nuclear fragment was removed.

**3.2 Surgical Protocols**

Surgery will be performed by a single surgeon (the PI, Dr. Liu).

**3.2.1 Surgical Protocol in the Traditional technique Group**

- A 3.0-mm temporal clear corneal incision was created.
- 5.5-mm capsulorhexis was made.
- IOL was planted in the bag after all nucleuses were removed.

**3.2.2 Surgical Protocol in the new technique Group**

- A 3.0-mm temporal clear corneal incision was created.
- 5.5-mm capsulorhexis was made.
- IOL was planted in the bag before the last nuclear fragment was removed.
- The last fragment was removed by phacoemulsification.

**3.3 Postoperative Medical Therapy**

For all patients, the standard postoperative regimen will consist of topical therapy included 0.3% tobramycin and 0.1% dexamethasone eye drops (Tobradex, Alcon Laboratories) four times per day and 0.3% tobramycin and 0.1% dexamethasone eye ointment (Tobradex, Alcon Laboratories) every night for one month.

**Chapter 4**

**Follow-up and evaluation of surgical outcomes**

**4.1 Follow-up Examination Schedule**

The frequency of follow-up examinations will approximate that practiced in standard clinical care, but may take place more frequently if there are complications. At a minimum, the patient will be examined 1day, 7 days, and 30 days following cataract surgery.

**4.2 Follow-up Examination Procedures**

Principal safety outcomes include four measurements: central corneal endothelial cell loss, central cornea thickness, temporal corneal incision thickness and posterior capsular rupture.

Intraoperative outcomes include five measurements: ultrasound time (UST), US total equivalent power in position 3 (US power), cumulative dissipated energy (CDE), total BSS volume (V_BSS_), and surgical complications, recorded at the end of each surgery.

Postoperative outcomes include four measurements: (1) The un-corrected visual acuity (UCVA) was recorded by an ETDRS chart preoperatively at postoperative day 1, 7 and 30. (2) The maximal incision thickness and (3) central corneal thickness were recorded by anterior segment optical coherence tomography preoperatively and at each postoperative visit. (4) Central corneal endothelial cell density was recorded preoperatively and at postoperative 7 and 30 day by non-contact specular microscopy.

**Chapter 5**

**Statistical Analyses**

The SPSS software package (version 17.0, SPSS Inc, Chicago, IL, USA) was used for statistical analysis. Changes of central corneal thickness and changes of temporal corneal incision thickness were compared using repeated measures ANOVA. Mean central cornea endothelial cell loss, UST, US power, CDE, and total BSS volume of the two groups were compared using a t test. Pearson’s chi-squared test was used for comparing gender, nucleus grade, Fisher’s exact test was used for preoperative UCVA and Mann-Whitney test was used for postoperative UCVA. Changes in outcomes were compared between treatments using a mixed effects model with covariate adjustment for baseline outcome. Mean changes and 95%CI derived from the mixed models are presented. All statistical tests were two-tailed with α= 0.05. A p value of < 0.05 was considered statistically significant.

**Chapter 6**

**References**

1. Wang D, Huang G, He M, Wu L, Lin S. Comparison of anterior ocular segment biometry features and related factors among American Caucasians, American Chinese and mainland Chinese. Clin Experiment Ophthalmol. 2012 Aug; 40(6):542-9.
2. Yan PS, [Lin HT](http://www.ncbi.nlm.nih.gov/pubmed?term=%22Lin%20HT%22%5BAuthor%5D) , [Wang QL](http://www.ncbi.nlm.nih.gov/pubmed?term=%22Wang%20QL%22%5BAuthor%5D), [Zhang ZP](http://www.ncbi.nlm.nih.gov/pubmed?term=%22Zhang%20ZP%22%5BAuthor%5D). Anterior Segment Variations with Age and Accommodation Demonstrated by Slit-lamp-adapted Optical Coherence Tomography. [Ophthalmology.](javascript:AL_get(this,%20'jour',%20'Ophthalmology.');) 2010. 117(12):2301-7.
3. He M, Huang W, Zheng Y, Alsbirk PH, Foster PJ. Anterior chamber depth in elderly Chinese: the Liwan eye study. Ophthalmology. 2008 Aug; 115(8):1286-90.
4. Zeng M, Liu X, Liu Y, Xia Y, Luo L, Yuan Z, Zeng Y, Liu Y. Torsional ultrasound modality for hard nucleus phacoemulsification cataract extraction. The British journal of ophthalmology. 2008. 92:1092-6.
5. Wang Y, Xia Y, Zeng M, Liu X, Luo L, Chen B, Liu Y, Liu Y. Torsional ultrasound efficiency under different vacuum levels in different degrees of nuclear cataract. Journal of cataract and refractive surgery. 2009. 35(11):1941-1945.
